# Introduction of inflammatory bowel disease biomarkers panel using protein-protein interaction (PPI) network analysis

**Published:** 2016-12

**Authors:** Hamid Asadzadeh-Aghdaee, Shabnam Shahrokh, Mohsen Norouzinia, Mostafa Hosseini, Aliasghar Keramatinia, Mostafa Jamalan, Bijan Naghibzadeh, Ali Sadeghi, Somayeh Jahani Sherafat, Mohammad Reza Zali

**Affiliations:** 1 *Basic and Molecular Epidemiology of Gastrointestinal Disorders Research Center, Research institute for Gastroenterologyand Liver Diseases, Shahid Beheshti University of Medical Sciences, Tehran, Iran*; 2 *Gastroenterology and Liver Diseases Research Center, Research Institute for Gastroenterology and Liver Diseases, Shahid Beheshti University of Medical Sciences, Tehran, Iran*; 3 *Faculty of Medicine, Iran University of Medical Sciences, Tehran, Iran*; 4 *Proteomics Research Center, Shahid Beheshti University of Medical Sciences, Tehran, Iran*; 5 *Abadan School of Medical Sciences, Abadan, Iran*; 6 *Hearing Disorders Research Center, Shahid Beheshti University of Medical Sciences, Tehran, Iran*; 7 *Department of Biomedical Engineering, Amirkabir University of Technology, Tehran, Iran*; 8 *Behbood Gastroenterology and Liver Diseases Research Center, Shahid Beheshti University of Medical Sciences, Tehran, Iran*

**Keywords:** Inflammatory bowel diseases (IBD), Protein-protein interaction (PPI) network analysis, Hub-bottlenecks, Protein clusters

## Abstract

**Aim:**

In the present study, a protein-protein interaction network construction is conducted for IBD.

**Background:**

Inflammatory bowel diseases as serious chronic gastrointestinal disorders attracted many molecular investigations. Diverse molecular information is present for IBD. However, these molecular findings are not highlighted based on interactome analysis. On the other hand, PPI network analysis is a powerful method for study of molecular interactions in the protein level that provide useful information for highlighting the desired key proteins.

**Methods:**

Cytoscape is the used software with its plug-ins for detailed analysis. Two centrality parameters including degree and betweenness are determined and the crucial proteins based on these parameters are introduced.

**Results:**

The 75 proteins among 100 initial proteins are included in the network of IBD. Seventy-five nodes and 260 edges constructed the network as a scale free network. The findings indicate that there are seven hub-bottleneck proteins in the IBD network.

**Conclusion:**

More examination revealed the essential roles of these key proteins in the integrity of the network. Finally, the indicator panel including NFKB1, CD40, TNFA, TYK2, NOD2, IL23R, and STAT3 is presented as a possible molecular index for IBD.

## Introduction

Inflammatory bowel diseases (IBD) are chronic gastrointestinal disorders, caused by a diysregulated immune response to host intestinal micro flora. The two principal types of inflammatory bowel disease are ulcerative colitis (UC), which is primarily restricted to the colon and rectum, and Crohn disease (CD), which can affect any segment of the gastrointestinal tract from the mouth to the anus. Individual’s life be affected with IBD and it has cost for the health care system and society (1- 3). Recent studies found that the incidence and prevalence of the diseases are still increasing (1, 3-5). The etiologies of IBD remain uncertain but genetic and environmental factors have the main role on establishing the diseases (6-9). Although clinical finding, laboratory tests and imaging could aid to establish the diagnosis but it is usually confirmed by biopsies on colonoscopy (10).

However, it may be difficult and time- consuming to make even for trained physicians (11). IBD can be associated with serious complications and may lead to aggressive processor. Patients with IBD are more prone to the development of malignancy. Persons with Crohn’s disease have a higher rate of small bowel malignancy (15). There are many reports about molecular aspects of IBD. One of them is based on protein level examination. Bioinformatics can be helpful to provide a new perspective of molecular changes in diseases such as IBD. One of the important disciplines in bioinformatics is protein-protein interaction (PPI) network analysis. In fact, proteins are in a complex interactome organization that any small changes in each individual may lead to dysfunction of the whole system (12). Topological characteristic in PPI network is a criterion for determination of the key elements of a network(13). Centrality is the major part of the topological characteristic of a PPI network. Many centrality parameters are defined for network analysis. However, some of them proved to be more informative than the other ones in prioritize of elements of a network (14). In this regard, degree and betweenness are the two more applied centrality parameters for network analysis. Proteins with high degree are known as hubs while proteins with high betweenness centrality are introduced as bottlenecks (15). On the other hand, proteins that show both features are called hub-bottleneck agents that are prominent in network integrity (16). In addition, PPI network consist of complexes of proteins in which are clusters of interconnected proteins playing crucial part in a network. These clusters contain seed proteins, which play the major role in functional aspects of a cluster (17). Therefore, PPI network evaluation and complex analysis of IBD essential proteins are important to provide a new glance of the disease.

## Material and Methods

One of the valuable sources for network construction is STRING Database, which is accessible through Cytoscape *3.4.0-Milestone 2*(18). String db has three options for providing information including protein query, PubMed query, and disease query. Here, DISEASE Database query was chosen for retrieving proteins related to STRING db provides interactome information from different sources such as experimental and text minding data with the related scores (19). STRING db, by the evaluation of these scores presents a combined disease score for the corresponding retrieved proteins. Additionally, confidence score that is estimated by the cut off in the query determines the validity of interactions. Here, the cutoff of 0.4 was set for the analysis as a default option. This score is scaled between 0 and 1. About 100 nodes from STRING Database were selected for construction of the network. Further analysis composed of topological parameters examination by the use of Network Analyzer plugin, which is well-integrated in Cytoscape Software. The evaluated parameters are degree and betweenness centrality (BC). The significance of these centrality parameters is that they show the prominent nodes in the network that are central for the network strength. Hub and bottlenecks are the terms used for proteins with large degree and high BC, respectively. The hub-bottleneck proteins are the most vital agents in a network. At first, the top proteins with high degree are introduced and then the proteins with the highest degree and BC values are considered as hub- bottlenecks. Moreover, the sub-network analysis of STAT3 as the top bottleneck element was performed to understand the behavior of this protein and its relationship with other nodes of the network. This network is constructed by determining the first neighbors of STAT3. Clustering analysis of the network was then handled by MCODE algorithm. This plug-in extracts the protein complexes that are imperative in a PPI network. The protein with highest interconnection is called the seed protein. Clusters are ranked based on their related scores, which is obtained by the interconnection determination. The prediction of clusters is based on vertex weighting by local neighborhood density and outward traversal from a locally dense seed protein to isolate the dense regions according to given parameters (17). It is known that proteins within specific clusters possess similar functions and are participated in individual biological process. The criteria for protein complex determination are as follows: Degree Cutoff: 2, Node Score Cutoff: 0.2 and Max Depth: 100.

## Results

**Figure 1:**
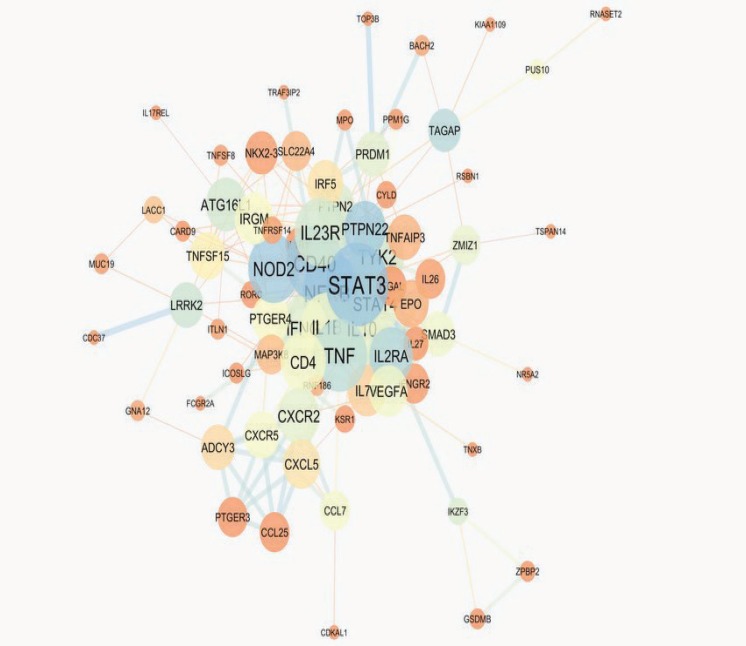
PPI network of IBD including 75 proteins and 260 edges. The initial proteins were 100 proteins but 19 proteins were isolated and 3 pairs were not included in network. The larger circles correspond to the higher degrees and Brown to blue color refers to increment of betweenness

**Figure 2:**
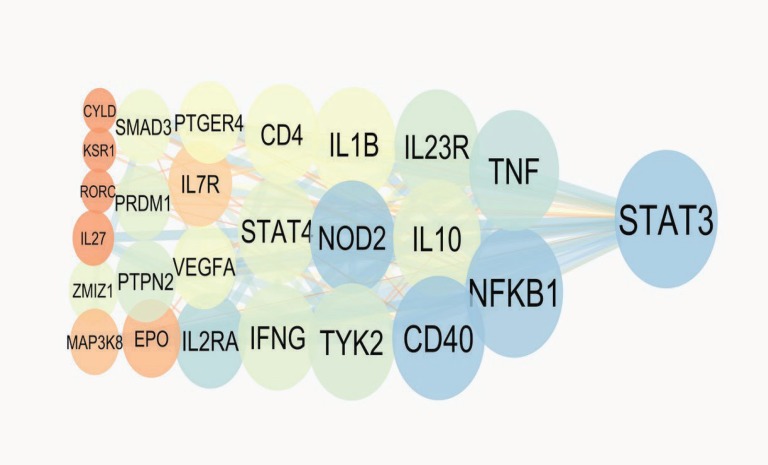
The direct linked proteins to STAT3. The larger circles correspond to the higher degrees and brown to blue color refers to increment of betweenness

The PPI network of IBD including 100 proteins is constructed and presented in figure 1. Nineteen proteins are not linked to the network and also 3 pairs are isolated. Therefore, 75 proteins among 100 are included in network. The top ten hub proteins are determined and tabulated in table 1. Based on BC≥0.05, 7 hub proteins are introduced as hub-bottlenecks proteins (see table 1). For more resolution, the direct connected proteins to the STAT3 (as the central protein in the network) are shown in figure 2. Four clusters of IBD network and their properties are tabulated in the table

**Figure 3:**
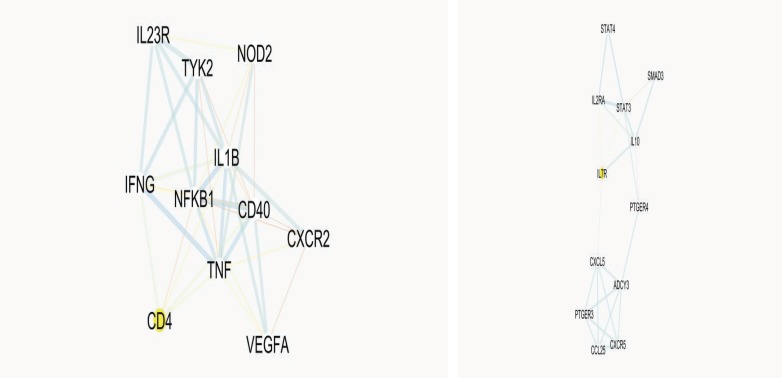
There are four clusters in the PPI network of IBD. Two clusters are important (clusters 1, up and 2, down) because seven key proteins (hub-bottlenecks) are organized in these clusters

2. The key proteins of the network are distributed in manner between the four clusters. Only two clusters contain the hub- bottlenecks proteins (see figure 3).

## Discussion

Protein-protien interaction analysis can provide useful information for many diseases such as diseases related to digestive system (14). We have chosen IBD as one of the important bowel diseases is candidated for PPI network analysis.There are several reported genes and proteins for IBD development conducted by many molecular investigations (20, 21). STRING db as a powerful application in Cytoscape combines linked proteins and their interaction data from different molecular sources (19). About 100 proteins were selected for this study by the cutoff 0.4. However, only 75 proteins showed contribution in the main network. The other 25 proteins were deleted because they were not interavtive with the main network. As it is depicted in figure1, 260 edges are organized for 75 nodes that there is about 3.5 edges for each node. Yet, the edges distribution is not homogeneous. In fact, the PPI network as a scale free map, nodes show different interactive behavior. This characteristic of the nodes is used for centrality ranking of them (22). The finding indicate that there are some nodes that can be differentiated from the others by the number of their links and short path pass through them. Quantitative calculation of these essential nodes is tabulated in table1. Top ten proteins in the IBD network, based on the degree are selected as hub nodes. Hub proteins are key proteins that show large values of interactions. Therefore, any changes in protein expression of these proteins in a network may conclude in deep dysfuction of the interactiome system (16). Bottleneck proteins are the nodes with high betweenness centrality values. Changes in protein expression of bottlenecks may also result in vast alteration in a network integrity (23). Some key protiens simultaneously are hub and bottleneck nodes. These proteins are absolutely the main proteins in a network. Here, these proteins are introduced in table1, in which seven proteins are found with these properties. Regarding disease scores, these hub-bottleneck proteins have significant association with IBD, in a way that two of them belong to the first four top scored nodes. It is expected that the relationship of these key proteins in IBD disease in literature is referred as potential biomarkers. Even not so, this panel of highlighted proteins purposes a new level of information for IBD that increases our knowledge about diagnostic and therapeutic aspects of disease. Nevertheless, validation studies are required in this regard. Furthermore, STAT3, as a potent key protein in IBD network, is connected directly to the all hub-bottlenecks as shown in figure 2 (24). This closed interactions between the central proteins confirms the novel introduced panel in this analysis. A network may consider as several connected clusters (14). In this analysis, there are four clusters retrived by MCODE (table2 and figure3) which the hub-bottleneck proteins are organized in only the first two of them. The importance of these two first clusters is that, the first one possesses the highest numbers of hub- bottleneck proteins and in the second contain STAT3 as our important top protein. The presents of the seven key proteins in these two clusters show their noteworthy values in the network topology. While the seed proteins of these clusters are not hub- bottlenecks but they play a major role in IBD network. It seems that the introduced panel may reflect the disease manifestation and development.

**Table 1:** Top ten elements of the analysis. The proteins are ranked based on degree values. By considering BC≥0.05 and the hub proteins are introduced as hub-bottlenecks (the correspond gene names are asterisked in the table). Four clusters are determined in the network and the key proteins are organized in the two clusters 1 and 2.

Row	Gene Name	Protein Name	Disease Score	Degree	BC	Cluster No.
**1**	*STAT3	signal transducer and activator of transcription 3 (acute-phase response factor)	3.98	25	0.11	2
**2**	*NFKB1	nuclear factor of kappa light polypeptide gene enhancer in B-cells 1	3.60	23	0.11	1
**3**	*CD40	CD40 molecule, TNF receptor superfamily member 5 tumor necrosis factor	3.76	21	0.11	1
**4**	* TNFA	necrosis factor	3.63	20	0.08	1
**5**	IL10	interleukin 10	4.30	19	0.04	2
**6**	*TYK2	tyrosine kinase 2	3.49	19	0.05	1
**7**	*NOD2 nucl	eotide-binding oligomerization domain containing 2	5.00	18	0.11	1
		interleukin 23 receptor				
**8**	*IL23R	interleukin 1, beta	5.00	18	0.06	1
**9**	IL1B	interferon, gamma	3.09	18	0.02	1
**10**	IFNG		4.03	16	0.04	1

**Table2:** Four clusters of PPI network of IBD. The seed proteins for the relative clusters are determined

**Cluster No.**	**Seed**	**Score**	**Hub-bottlenecks**
1	CD4	7.6	NFKB1, CD40, TNFA, TYK2, NOD2, IL23R
2	IL7R	4.36	STAT3
3	TNFSF15	4	-
4	IKZF3	3	-

According to the other complex polygenic and multifactorial disease, the PPI network panels beside molecular mechanisms, environment and gut microbiota lead to multidimensional sequential panel to access personalized medicine in IBD patients (25).

In conclusion, the seven ranked nominated proteins in this research as a suitable indicator panel may have a possible role in clinical usage and managements for IBD disease. It is suggested that this purposed panel to be assessed in the field by the application of the related chip.
